# Supplementary material for: A Prospective, Randomized, Placebo-Controlled Study Assessing the Efficacy of Chinese Herbal Medicine (Huangqi Guizhi Wuwu Decoction) in the Treatment of Albumin-Bound Paclitaxel-Induced Peripheral Neuropathy
Source: J Clin Med. 2023 Jan 7;12(2):505. doi: 10.3390/jcm12020505 (PMC9863646; doi:10.3390/jcm12020505)
Supplement: Supplementary file 1 [file jcm-12-00505-s001.zip › jcm-2088224-supplementary.pdf]

Supplemental Table S1. QLQ-CIPN20 items (1) (2)

1. Did you have tingling fingers or hands? [a]
2. Did you have tingling toes or feet? [a]
3. Did you have numbness in your fingers or hands? [a]
4. Did you have numbness in your toes or feet? [a]
5. Did you have shooting or burning pain in your fingers or hands? [a]
6. Did you have shooting or burning pain in your toes or feet? [a]
7. Did you have cramps in your hands? [b]
8. Did you have cramps in your feet? [b]
9. Did you have problems standing or walking because of difficulty feeling the ground under your feet? [a]
10. Did you have difficulty distinguishing between hot and cold water? [a]
11. Did you have a problem holding a pen, which made writing difficult? [b]
12. Did you have difficulty manipulating small objects with your fingers (for example, fastening small buttons)? [b]
13. Did you have difficulty opening a jar or bottle because of weakness in your hands? [b]
14. Did you have difficulty walking because your feet dropped downwards? [b]
15. Did you have difficulty climbing stairs or getting up out of a chair because of weakness in your legs? [b]
16. Were you dizzy when standing up from a sitting or lying position? [c]
17. Did you have blurred vision? [c]
18. Did you have difficulty hearing? [a]
19. Please answer the following question only if you drive a car: Did you have difficulty using the pedals? [b]
20. Please answer the following question only if you are a man: Did you have difficulty getting or maintaining an erection? [c]

Items have been renumbered from the original QLQ-CIPN20 instrument

[a] Sensory scale items

[b] Motor scale items

[c] Autonomic scale items

References:

1. Postma TJ, Aaronson NK, Heimans JJ, Muller MJ, Hildebrand JG, Delattre JY, et al. The development of an EORTC quality of life questionnaire to assess chemotherapy-induced peripheral neuropathy: the QLQ-CIPN20. *Eur J Cancer* (2005) 41(8):1135-9. doi: 10.1016/j.ejca.2005.02.012. PubMed PMID: 15911236.
2. Lavoie SE, Barton DL, Qin R, Steen PD, Aaronson NK, Loprinzi CL. Assessing patient-reported peripheral neuropathy: the reliability and validity of the European Organization for Research and Treatment of Cancer QLQ-CIPN20 Questionnaire. *Qual Life Res* (2013) 22(10):2787-99. doi: 10.1007/s11136-013-0379-8. PubMed PMID: 23543373.
